# Effects of a Cu_*x*_O Buffer Layer on a SiO_*x*_-Based Memory Device in a Vaporless Environment

**DOI:** 10.1186/s11671-015-1003-3

**Published:** 2015-07-14

**Authors:** Chih-Yi Liu, Zheng-Yao Huang

**Affiliations:** Department of Electronic Engineering, National Kaohsiung University of Applied Sciences, No.415, Chien Kung Road, Kaohsiung, 807 Taiwan

**Keywords:** Cu_*x*_O, Resistive switching, SiO_*x*_, 73.50.-h, 73.40.Rw, 73.61.Ng

## Abstract

The resistive switching characteristics of the Cu/SiO_*x*_/Pt structure (control sample) exhibited a direct correlation to humidity. The H_2_O vapor formed the Cu oxide at the Cu/SiO_*x*_ interface, and Cu ions were injected from the Cu oxide into the SiO_*x*_ layer, thus improving the resistive switching. However, the control sample demonstrated substantial switching dispersion in a vaporless environment. The Cu_*x*_O layer in the Cu/Cu_*x*_O/SiO_*x*_/Pt structure (Cu_*x*_O sample) helped the dissolution of Cu ions from the Cu electrode into the SiO_*x*_ layer, enabling effective electrochemical resistive switching in a vaporless environment. The Cu_*x*_O sample exhibited low switching dispersion and favorable endurance characteristics in a vaporless environment.

## Background

Recently, resistive random access memory (RRAM) has attracted considerable interest because of its non-volatile resistance change in simple metal/insulator/metal structures [1, 2]. Depending on the material group [3], device structure [4, 5], and defect status [6, 7], various switching behaviors can be observed. The switching mechanisms are typically classified as either a valence change effect [8], a thermochemical effect [2, 9], or an electrochemical effect [10, 11], whereby distinct surface effects can be attributed to various mechanisms [12, 13]. Ke et al. proposed that the oxygen concentration influences the redox reaction in ZnO resistive switching [12], and Tsuruoka et al. suggested that H_2_O vapor plays as an essential role in the redox reaction of an electrochemical Cu/SiO_2_/Pt device [13, 14]. The effects of moisture on Cu/SiO_2_/Pt and Cu/Ta_2_O_5_/Pt devices were different due to different adsorption coefficients of water. H_2_O vapor formed a Cu oxide interface between the Cu electrode and the oxide layer after the forming process and also enhanced Cu migration within the oxide layer. The H_2_O vapor facilitates in the dissolution of Cu ions into the SiO_2_ layer, enabling effective resistive switching. The Cu/SiO_2_/Pt device does not perform the resistive switching without H_2_O vapor [13, 14]. However, there is no H_2_O vapor within a packaged chip, and this is a serious concern, because these devices are used in memory applications.

In this study, a Cu_*x*_O layer was inserted between the Cu and SiO_*x*_ layer to improve the electrochemical resistive switching and minimize switching dispersion in a vaporless environment.

## Methods

**Fig. 1:**
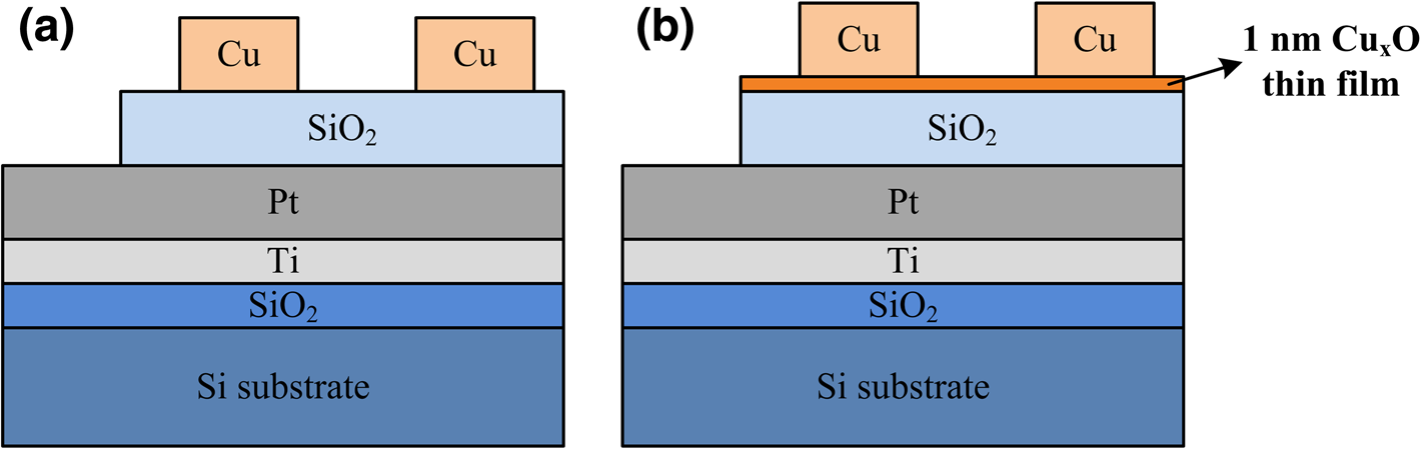
Illustrations of the sample structures. **a** the control sample and **b** the Cu_*x*_O sample

A 20-nm-thick SiO_*x*_ layer was deposited on a Pt-coated substrate (Pt/Ti/SiO_2_/Si) using radio frequency sputtering at room temperature. Subsequently, a 200-nm-thick Cu electrode was deposited using a thermal evaporator at room temperature, to form the final Cu/SiO_*x*_/Pt structure (control sample). The device area was 5 × 10^−5^ cm^2^. To create the Cu/Cu_*x*_O/SiO_*x*_/Pt structure (Cu_*x*_O sample), an additional 1-nm Cu_*x*_O layer was deposited on the SiO_*x*_ layer by using a thermal evaporator at room temperature. The structures of the control sample and the Cu_*x*_O sample are illustrated in Fig. 1, respectively. X-ray photoelectron spectroscopy (XPS; PHI-5000, ULVAC-PHI) was used to analyze the composition of the Cu_*x*_O layer. The electrical measurements were performed using an HP 4155B semiconductor parameter analyzer, and the measurements were taken in both air (60 % relative humidity) and N_2_ environments at room temperature. The bias voltage was applied on the top electrode while the bottom electrode was grounded.

## Results and Discussion

**Fig. 2:**
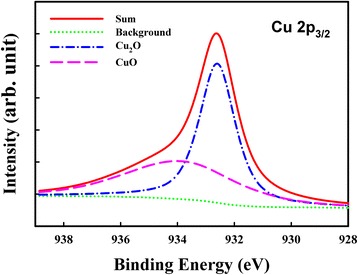
XPS of the Cu_*x*_O layer. Cu 2p_3/2_ XPS spectra of the Cu_*x*_O layer

**Fig. 3:**
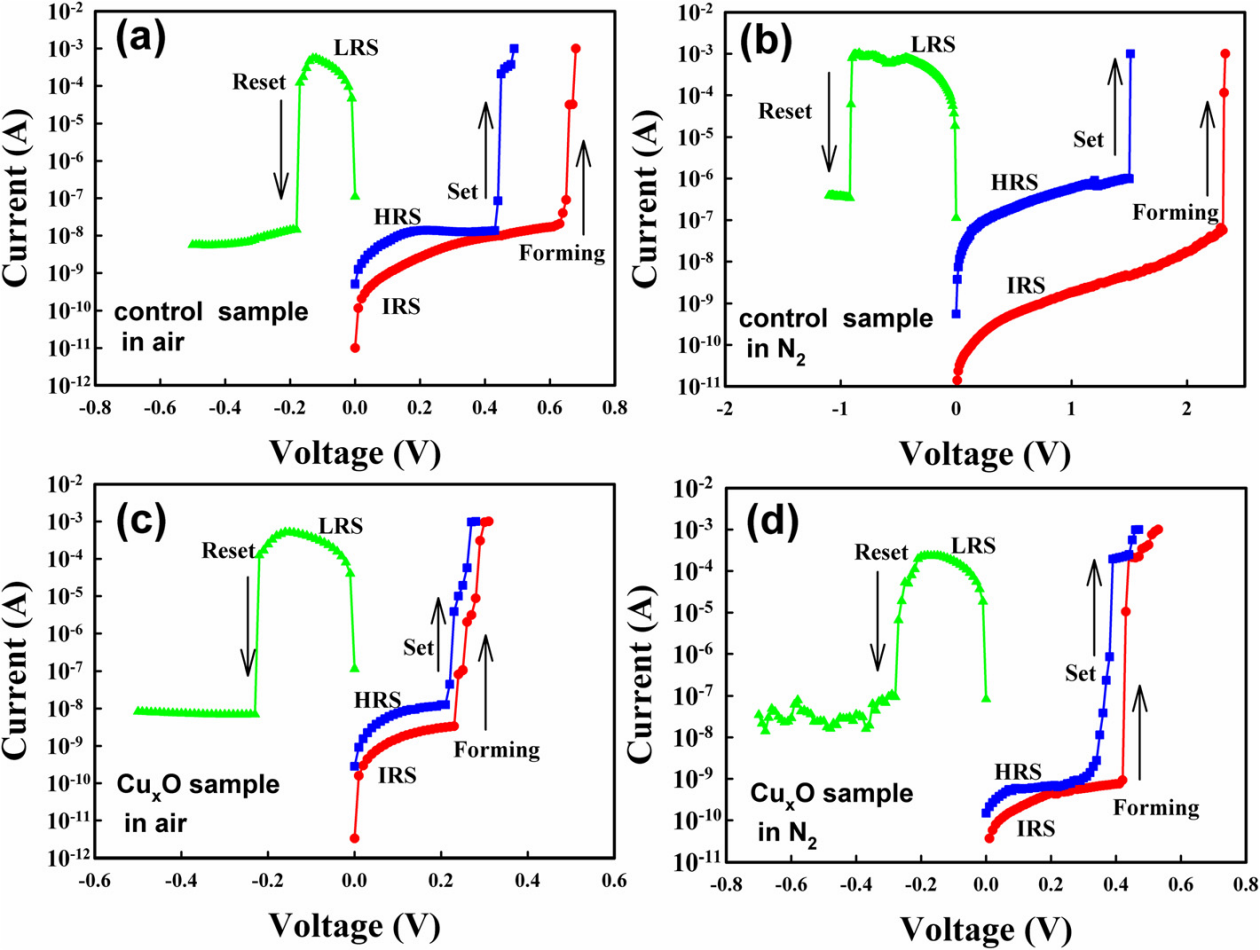
The resistive switching characteristics in different measurement environments. **a** The control sample in air. **b** The control sample in N_2_. **c** The Cu_*x*_O sample in air. **d** The Cu_*x*_O sample in N_2_

Figure 2 depicts the XPS Cu 2p_3/2_ spectra of the Cu_*x*_O layer. The detailed chemical structure information was extracted through deconvolution of Cu 2p_3/2_ peaks, which showed a CuO peak locating at 934 eV and a Cu_2_O peak locating at 932.6 eV. The Cu_*x*_O layer is the mixture of CuO and Cu_2_O. Figure 3 shows the resistive switching characteristics of the control and the Cu_*x*_O samples in both air and N_2_ environments. A positive-forming voltage was used to initialize the resistive switching; the resistance state was then switched from an initial resistance state (IRS) to a low resistance state (LRS). Following this transition, a negative Reset voltage was applied to switch from the LRS to a high resistance state (HRS). Subsequently, using a positive Set voltage, the HRS was switched back to a LRS. The device resistance can be reversibly switched between a LRS and a HRS by a Set voltage and a Reset voltage. Figure 3c, d illustrates the resistive switching characteristics of the Cu_*x*_O sample in air and N_2_ environments, and the behaviors were similar to those of the control sample in air. According to our previous study [15], device structure, and switching behavior, the resistive switchings of the control sample in air and the Cu_*x*_O sample in both environments are dominated by the electrochemical model with a Cu conducting filament. However, as shown in Fig. 3b, the control sample in the N_2_ environment required substantially higher operating voltages. The control sample in the N_2_ environment also showed a reversible unipolar switching, and the temperature coefficient of LRS resistance was negative. Therefore, the switching mechanism should be the thermochemical effect with conducting filaments of oxygen-related defects.

**Fig. 4:**
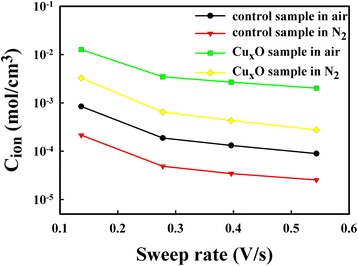
Influence of the measurement environment on the Cu ion concentration. The Cu ion concentrations of the control sample and the Cu_*x*_O sample in air and N_2_

Tsuruoka et al. proposed that a Cu/SiO_2_/Pt structure exhibits no resistive switching in a vacuum or N_2_ environment (no H_2_O vapor) because of the desorption of residual water from the SiO_2_ layer [13]. Because the composition of the SiO_*x*_ film influences the switching mechanism [16], the result of this study in N_2_ differed from the result of Tsuruoka et al., which may be due to the dissimilar composition between SiO_*x*_ and SiO_2_ layers. Although there was some difference between Tsuruoka’s study and our control sample in N_2_, these two samples both performed unfavorably because of a lack of humidity and exhibited no electrochemical resistive switching. Because no copper oxide was formed at the Cu/SiO_*x*_ (or SiO_2_) interface of these two samples, no electrochemical resistive switching was observed. The Cu ion concentrations were calculated using the cyclic voltammetry (CV) method [17], and the results are presented in Fig. 4. The CV method is used to study the redox reaction of Cu within the device. The applied voltage swept to 0.15 V and then swept back until the current was zero. During this sweeping period, oxidized Cu ions were injected into the SiO_*x*_ thin film. The amount of Cu ions (*Q*) can be calculated, and then, Cu ion concentration (*C*_ion_) can be determined by the formula ${C}_{\mathrm{ion}}=\frac{Q}{q{N}_A}/V$, where *N*_*A*_ is Avogadro’s number and *V* is the device volume. The Cu_*x*_O sample contained higher Cu ion concentrations than those in the control samples. In addition, the two samples contained larger Cu ion concentrations in air than in N_2_. Tsuruoka et al. proposed that H_2_O molecules would form a hydrogen-bond network at grain boundaries in SiO_2_ [13] and thus had larger Cu ion concentration. Therefore, the humidity and Cu_*x*_O layer both help the dissolution of Cu ions into SiO_*x*_ layer. The electrochemical resistive switching has three rate-limiting processes [13]: the Cu ionization at the Cu/SiO_*x*_ interface, the migration of Cu ions in the SiO_*x*_ film, and the nucleation of Cu at the Pt bottom electrode. In this study, the Cu nucleation at Pt electrode would not the rate-limiting process. The control sample in N_2_ had the lowest Cu ion concentrations, and it cannot show an electrochemical resistive switching, which means that the Cu ionization at the Cu/SiO_*x*_ interface is the rate-limiting process in N_2_. Willis and Lang [18] proposed the three possible mechanisms for the Cu ionization at the Cu/SiO_2_ interface under a positive applied voltage: the anodization-like process that Cu atoms are injected directly into the SiO_2_ layer by the dissolution reaction Cu → Cu^*z*+^ + *ze*^−^, Cu ions injected into the SiO_2_ from the Cu_*x*_O formed at the Cu/SiO_2_ interface due to reduction of the SiO_2_, and the chemical oxidation of Cu atoms at the Cu/SiO_2_ interface via H_2_O, O_2_, or out-gassing of the oxide [13]. In this study, the Cu/SiO_*x*_/Pt cannot electrochemically switch in N_2_. Therefore, the former two mechanisms should be excluded.

**Fig. 5:**
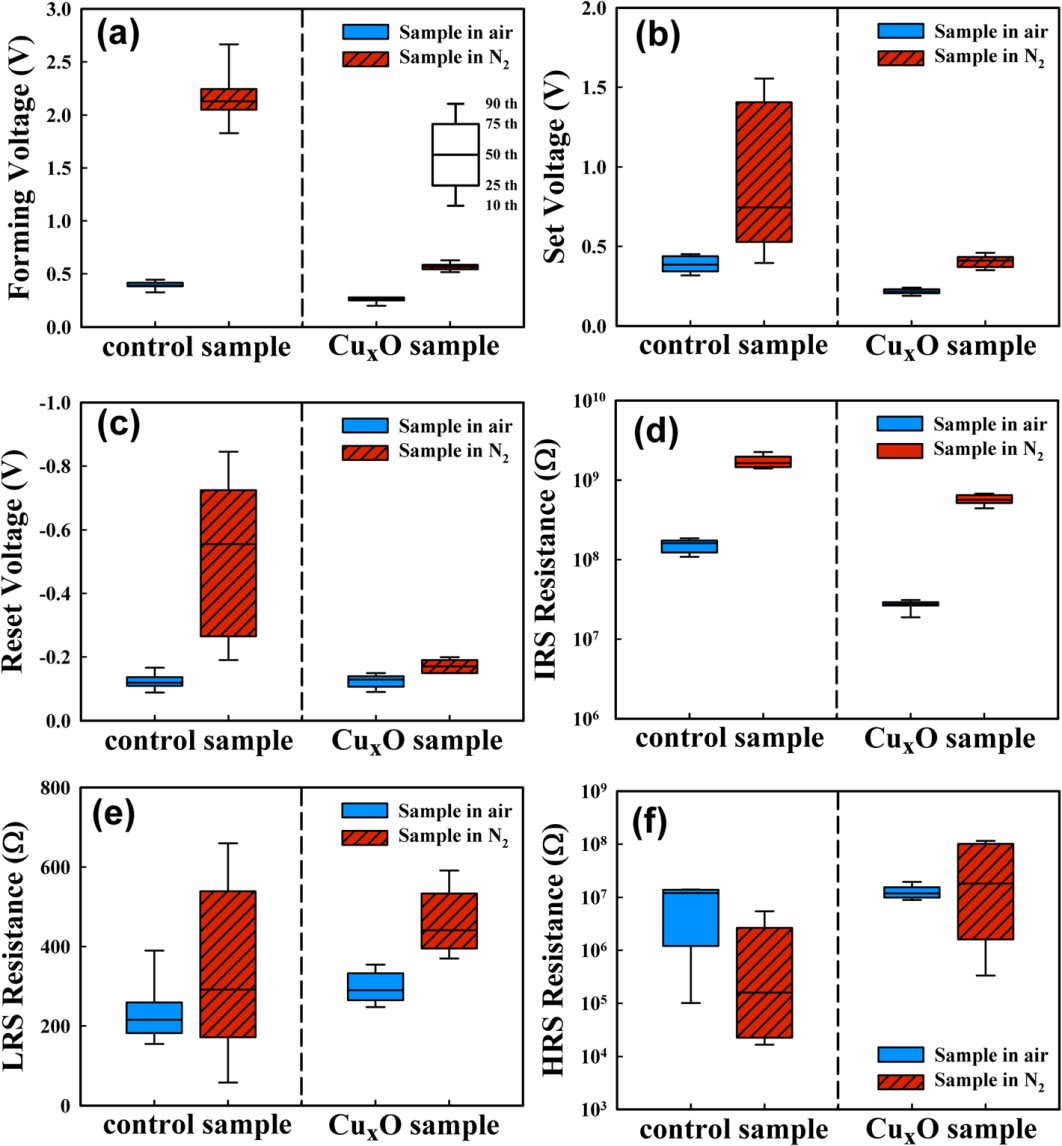
The parameters of resistive switching of the two samples. **a** The forming voltage. **b** The Set voltage. **c** The Reset voltage. **d** The IRS voltage. **e** The LRS resistance. **f** The HRS resistance

Figure 5a–c depicts the operating voltages of the two samples in both environments. The control sample in N_2_ exhibited the highest operating voltages because of the lowest Cu ion concentration within the SiO_*x*_ layer. Both samples exhibited lower operating voltages in air, as the H_2_O vapor increased the Cu ion concentration in the SiO_*x*_ layer. Tappertzhofen et al. proposed that there was no copper oxide at Cu/SiO_2_ interface before the forming process, but the copper oxide was found after the forming process in air [14]. In addition, this electrochemical oxidation was not observed in anhydrous atmosphere. We also fabricated a Ni (200 nm)/Cu_*x*_O (1 nm)/SiO_*x*_ (20 nm)/Pt structure (not shown) which did not have an electrochemical resistive switching. This means that the 1-nm Cu_*x*_O interlayer did not provide enough Cu ions to form Cu conducting filaments. Since the Cu electrode cannot be directly ionized to be Cu ions and a 1-nm Cu_*x*_O interlayer does not provide enough Cu ions to form Cu conducting filaments, the possible explanation is that the Cu metal is transformed into immediate states of Cu oxide via the Cu_*x*_O layer and then Cu ions can be injected from these immediate states by a positive electric field. Since the copper oxide cannot be electrochemically formed without a moisture environment, a Cu_*x*_O layer in this study was deposited between the Cu and SiO_*x*_ layers to solve this issue. The deposited Cu_*x*_O layer also can help the dissolution of Cu ions from the Cu electrode into the SiO_*x*_ layer, thus increasing the Cu ion concentration in the SiO_*x*_ layer. This increase in the Cu ion concentration effectively reduced the forming and Set voltages. Figure 5d–f shows the device resistances in both air and N_2_. The LRS conduction of the two samples in air and N_2_ exhibited ohmic behavior. The LRS resistances were approximately determined according to the current compliance during the Set process. The two samples exhibited lower LRS resistances in air, which may be due to an increased Cu ion concentration in the SiO_*x*_ layer during the Set process [8]. This increased concentration of Cu ions caused a formation of wider conduction filaments. The IRS and HRS conductions of the two samples were dominated by the Schottky emission (not shown). The two samples exhibited lower IRS resistances in air because of a lower Schottky barrier height. All switching parameters of the Cu_*x*_O sample had larger variation in N_2_ than in air. H_2_O molecules would form a hydrogen-bond network at grain boundaries in SiO_2_ [13] and thus enhanced Cu migration in the SiO_*x*_ layer. Therefore, the Cu_*x*_O sample had larger Cu ion concentration in air than in N_2_. The supply of Cu ions influenced the operating voltages and the stability to grow Cu conducting filaments. Hence, the Cu_*x*_O sample had a lower Cu ion concentration in N_2_ and thus had larger variation in switching parameters.

**Fig. 6:**
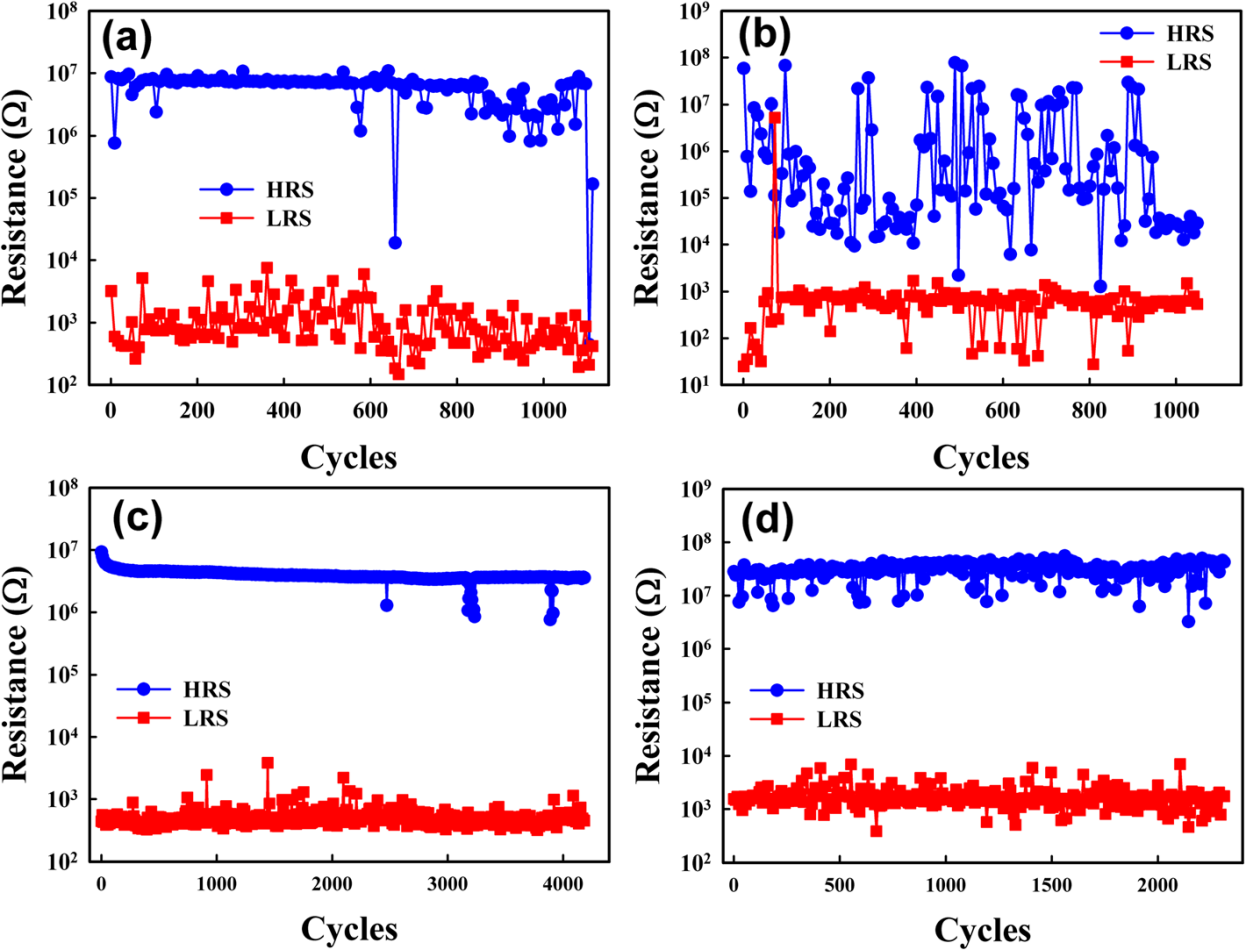
Endurance characteristics in different measurement environments. **a** The control sample in air. **b** The control sample in N_2_. **c** The Cu_*x*_O sample in air. **d** The Cu_*x*_O sample in N_2_

Figure 6 shows the endurance characteristics of the two samples in both environments. The control sample in N_2_ exhibited the least favorable endurance characteristics, because of its non-optimized thermochemical switching. Both of the samples exhibited a more favorable endurance in air than in N_2_, which indicated that H_2_O vapor increased the Cu ion concentration and thus improved the resistive switching. In a packaged chip, the RRAM device must switch in an environment without H_2_O vapor. The Cu_*x*_O sample demonstrated favorable endurance for more than 2000 cycles in the N_2_ environment, demonstrating that it is suitable for RRAM applications.

## Conclusions

This study investigated the addition of a Cu_*x*_O layer to a Cu/SiO_*x*_/Pt structure for maintaining the electrochemical resistive switching capabilities in a vaporless environment. The Cu ionization at the Cu/SiO_*x*_ interface is the rate-limiting process for the electrochemical resistive switching in N_2_. Therefore, Cu/SiO_*x*_/Pt cannot have an electrochemical resistive switching in N_2_. Therefore, in the Cu/Cu_*x*_O/SiO_*x*_/Pt structure, the Cu_*x*_O layer helped the dissolution of Cu ions from the Cu electrode into the SiO_*x*_ layer, effectively minimizing the switching dispersion. The Cu/Cu_*x*_O/SiO_*x*_/Pt also exhibited favorable endurance characteristics in a vaporless environment, demonstrating that it is suitable for practical applications.

## Abbreviations

HRS: high resistance state
IRS: initial resistance state
LRS: low resistance state
RRAM: resistive random access memory
XPS: X-ray photoelectron spectroscopy
